# Robust ferromagnetism carried by antiferromagnetic domain walls

**DOI:** 10.1038/srep42440

**Published:** 2017-02-14

**Authors:** Hishiro T. Hirose, Jun-ichi Yamaura, Zenji Hiroi

**Affiliations:** 1 Institute for Solid State Physics, University of Tokyo, Kashiwa, Chiba 277-8581, Japan; 2 Material Research Center for Element Strategy, Tokyo Institute of Technology, Yokohama, Kanagawa, 226-8503, Japan

## Abstract

Ferroic materials, such as ferromagnetic or ferroelectric materials, have been utilized as recording media for memory devices. A recent trend for downsizing, however, requires an alternative, because ferroic orders tend to become unstable for miniaturization. The domain wall nanoelectronics is a new developing direction for next-generation devices, in which atomic domain walls, rather than conventional, large domains themselves, are the active elements. Here we show that atomically thin magnetic domain walls generated in the antiferromagnetic insulator Cd_2_Os_2_O_7_ carry unusual ferromagnetic moments perpendicular to the wall as well as electron conductivity: the ferromagnetic moments are easily polarized even by a tiny field of 1 mT at high temperature, while, once cooled down, they are surprisingly robust even in an inverse magnetic field of 7 T. Thus, the magnetic domain walls could serve as a new-type of microscopic, switchable and electrically readable magnetic medium which is potentially important for future applications in the domain wall nanoelectronics.

Domain wall nanoelectronics puts the spotlight on a new paradigm of ferroic devices^1^. For example, the magnetic racetrack memory uses mobile domain walls in a ferromagnetic nanowire to store information^2^, and conducting domain walls in the ferroelectric insulator BiFeO_3_ are proposed to be used for a local strain sensor and a memory device^3^,^4^. Thus far, the target elements of the domain wall nanoelectronics have been limited to domain walls in ferroic orders. However, those in antiferroic orders such as antiferromagnetic order are also attractive because of the superior stability: domain walls in antiferroic orders can be stable even in the nanometer scale and robust against external fields in the absence of macroscopic polarizations. Theoretically predicted for magnetic domain walls (MDWs) in antiferromagnetic orders is the presence of uncompensated magnetic moments at the interface^5^, which has been actually observed in the specific case of the antiferromagnetic iron monolayer on the tungsten (001) substrate by means of spin-polarized scanning tunneling microscopy^6^. Such a MDW carrying a net magnetic moment may be useful as a magnetic recording medium. Thus, expanding the target to antiferroic orders would provide us with a chance to find a new route to microscopic devices with novel functionality in the domain wall nanoelectronics.

Recently, the MDWs in the all-in/all-out (AIAO) type antiferromagnetic order found in the two pyrochlore oxides Cd_2_Os_2_O_7_^7^ and *Ln*_2_Ir_2_O_7_ (*Ln* = Y and lanthanoids)^8^ draw attention as they show interesting physical properties. In these compounds, 5*d* electrons are conducting at high temperatures, while are completely localized at low temperatures to induce AIAO type magnetic orders; the metal-insulator (MI) transitions and magnetic orderings occur almost simultaneously at ~227 K in the osmate^9^,^10^ and at 34–110 K in the iridates^11^,^12^. The crystal structure of Cd_2_Os_2_O_7_ is shown in Fig. 1a, which contains a pyrochlore lattice made of corner-sharing tetrahedra of Os atoms. In the AIAO order, all the four Os magnetic moments of every tetrahedron point in or out to its center and cancel out in total. These two spin structures, “all-in” and “all-out”, alternate from one tetrahedron to its neighbors, as shown in Fig. 1b. There are two kinds of magnetic domains that can coexist^13^: the AIAO and all-out/all-in (AOAI) orders related by the time reversal symmetry with each other. The coexistence of the two domains has actually been visualized in Cd_2_Os_2_O_7_ by the circular polarized resonant X-ray diffraction imaging technique^14^. On the other hand, microwave impedance microscopy successfully visualized the MDWs of Nd_2_Ir_2_O_7_, which indicates that the MDWs remain conducting even when the domains themselves become insulating^15^,^16^. Similar conducting MDWs have also been suggested in Cd_2_Os_2_O_7_^10^,^17^. Interestingly, weak ferromagnetism is observed below *T*_N_ in both compounds^10^,^11^; the origin remains unclear and we speculate it related to the MDWs.

## Results

**Figure 1:**
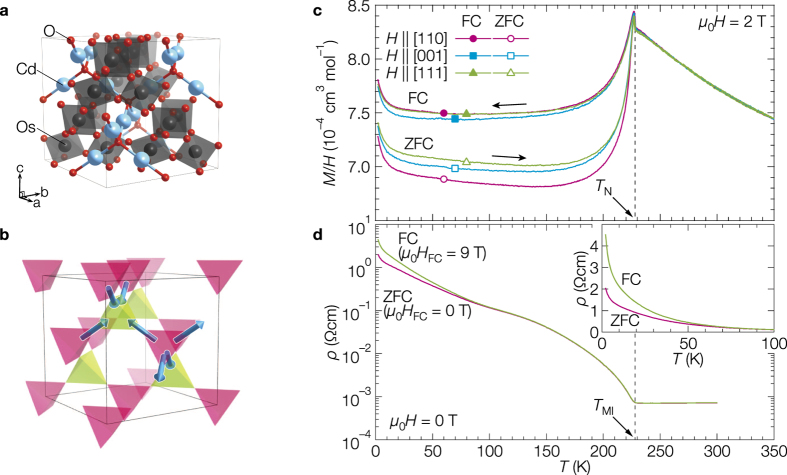
All-in/all-out (AIAO) order and temperature dependences of magnetic susceptibility and resistivity. (**a**) Crystal structure of Cd_2_Os_2_O_7_. Cadmium, osmium and oxygen atoms in the cubic unit cell of 10.1618(8) Å in edge are shown by cyan, black and red spheres, respectively. Four Os atoms, each of which is octahedrally coordinated by six oxygen atoms, form a regular tetrahedron which shares vertices with the surrounding four tetrahedra, resulting in the three-dimensional network of the pyrochlore lattice. (**b**) AIAO magnetic order on the pyrochlore lattice made of osmium atoms. The arrows represent some osmium spins on the vertices of tetrahedra; the tetrahedra with the all-in and all-out configurations are colored in yellow-green and magenta, respectively. (**c**) Temperature dependences of *M*/*H* measured on a single crystal of Cd_2_Os_2_O_7_. After cooling down to 2 K under zero magnetic field, magnetization was measured upon heating at a magnetic field of 2 T (ZFC) and then measured upon cooling at the same field (FC). The magnetic field was applied along the [110], [001], or [111] direction. (**d**) Temperature dependences of resistivity measured on a single crystal of Cd_2_Os_2_O_7_. The measurements were done at zero magnetic field upon heating after cooling to 2 K in *μ*_0_*H*_FC_ = 0 (ZFC) and 9 T (FC) along [111]. Electrical currents run along [1
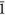
0], perpendicular to the cooling field. Inset shows an expanded view of the low temperature region in the linear scale.

In order to investigate the properties of the AIAO order and its MDWs in Cd_2_Os_2_O_7_, we have carried out precise magnetization *M* and resistivity *ρ* measurements under a various magnetic field *H* on bulk single crystals. The magnetic susceptibility *M*/*H* exhibits a Curie-Weiss-like temperature dependence at high temperatures followed by a distinct anomaly at *T*_N_ = 228 K (Fig. 1c), below which *ρ* rapidly increases (Fig. 1d). Below *T*_N_, there is a difference between the two *M*/*H* curves measured upon heating under 2 T after zero-field cooling (ZFC) and successively upon cooling under the field (FC). This feature is typical for an antiferromagnetic order accompanied by weak ferromagnetism^18^; ferromagnetic moments of domains freeze in random direction in ZFC, while they are forced to align along the applied *H* upon cooling across *T*_N_ in FC, so that the latter gives a larger *M*.

**Figure 2:**
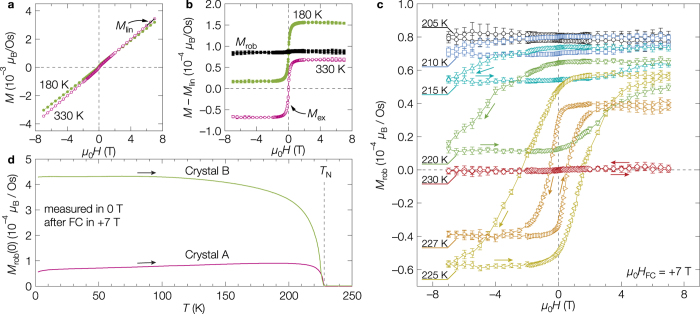
Magnetic field and temperature dependences of magnetization of Cd_2_Os_2_O_7_. (**a**) Magnetization curves at 180 and 330 K. First, crystal A was cooled from 350 K to the temperature in a magnetic field of *μ*_0_*H*_FC_ = +7 T along the [111] direction, and then the magnetization was measured with decreasing field to −7 T and with increasing field to +7 T. The black straight line on the 330 K curve indicates a linear component *M*_lin_ derived from a linear fitting at 6–7 T. (**b**) Magnetic field dependences of the non-linear component, *M* − *M*_lin_. The ferromagnetic component observed at 330 K is almost temperature independent, which is denoted as *M*_ex_. *M*_rob_ at 180 K is a residual ferromagnetic component emerged after further subtracting *M*_ex_, which is robust with almost no change in the magnetic field sweep between ±7 T. (**c**) Temperature evolution of *M*_rob_ between 205–230 K. The magnetization curves were measured sequentially from low to high temperatures after cooling to 205 K in *μ*_0_*H*_FC_ = +7 T. (**d**) Temperature dependences of remanent robust magnetizations [*M*_rob_(0)] on crystals A and B, which were measured in zero field upon heating after cooling to 2 K under +7 T.

Figure 2a shows magnetization curves at two typical temperatures above (330 K) and below *T*_N_ (180 K). In each curve, there are small non-linear components near the origin in addition to a large linear component *M*_lin_ from a paramagnetic or an antiferromagnetic state. Appeared after the subtraction of *M*_lin_ is a magnetization curve that is characteristic to a soft ferromagnet at 330 K, while an almost identical curve parallelly shifted upward at 180 K (Fig. 2b). The ferromagnetic component *M*_ex_ at 330 K is present above *T*_N_ and is almost temperature independent even across *T*_N_, which means that it must be irrelevant to the AIAO order (Supplementary Section 1). After subtracting *M*_lin_ and *M*_ex_ from the raw data at 180 K, there remains a nearly flat *M* independent of applied *H*; we call this robust magnetization *M*_rob_. Note that the magnitude of *M*_rob_ is less than 0.1% of the magnetic moment of osmium, *μ*_Os_ = 1–1.5 *μ*_B_, derived from ^17^O NMR experiments (Yamauchi, I. & Takigawa, M. in preparation).

The temperature evolution of *M*_rob_ near *T*_N_ is shown in Fig. 2c. The *M*_rob_ is almost *H* independent at 205 K as at 180 K, while it gradually opens a hysteresis loop and simultaneously moves downward with increasing temperature above 210 K. At 225 K, the *M*_rob_ eventually shows a typical behavior expected for a conventional ferromagnet; a symmetrical hysteresis loop with a complete sign change in the reversed field. Upon further heating, the hysteresis loop gradually shrinks, but still survives at 227 K, just 1 K below *T*_N_. Then, it completely disappears at 230 K. The temperature dependence of the remanent robust magnetization *M*_rob_(0) shown in Fig. 2d, which was measured in zero field after cooling to 2 K in +7 T, is almost constant at low temperatures, gradually decreases in approaching *T*_N_, and vanishes above *T*_N_. Therefore, it is obvious that the *M*_rob_ is a weak ferromagnetic moment “parasitic” to the AIAO order.

**Figure 3:**
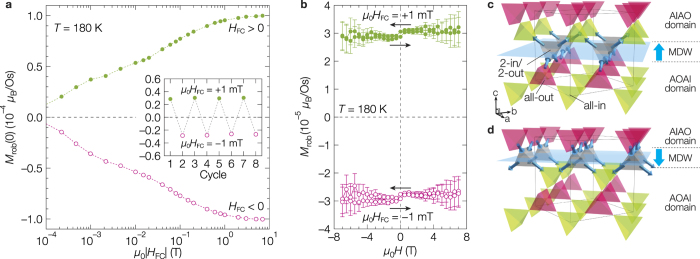
Robust ferromagnetic moment (*M*_rob_). (**a**) Remanent robust magnetization [*M*_rob_(0)] at 180 K as a function of *H*_FC_. Each *M*_rob_(0) was measured after cooling from a high temperature above *T*_N_ down to 180 K under various *H*_FC_ along [111]. Inset demonstrates the reproducibility of *M*_rob_(0) in a thermal cycle with alternating *μ*_0_*H*_FC_ = ±1 mT. (**b**) Magnetic field dependences of *M*_rob_ at 180 K measured after FC in ±1 mT. (**c**,**d**) Possible spin configurations at (001) MDWs in the classical picture. The MDW consists of tetrahedra each having the 2-in/2-out configuration (gray), in which two osmium spins point in and the other two point out so that an uncompensated magnetic moment perpendicular to the wall plane remains. All the uncompensated moments are forced to align upward in (**c**) whereas downward in (**d**) when the MDW moves up or down by one tetrahedron layer, as illustrated by large arrows. Spin arrangements realized with *H*_FC_ along the [001] and [00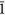] directions must be (**c**,**d**) respectively.

Although the *M*_rob_ is insensitive to *H* at low temperatures, it is highly sensitive to the cooling magnetic field *H*_FC_ which is applied along the [111] direction while the crystal is cooled across *T*_N_. The *H*_FC_ dependence of *M*_rob_(0) at 180 K is shown in Fig. 3a. Even in subtle *μ*_0_*H*_FC_ as low as ±1 mT, the *M*_rob_ changes its sign and takes a value corresponding to ~1/3 of its saturation along *H*_FC_. In sharp contrast, the *M*_rob_ never changes at 180 K even in the large reverse *H* of ±7 T (Fig. 3b). Furthermore, the magnitude of *M*_rob_ is determined only by the *H*_FC_ and takes nearly the same values in a thermal cycle as shown in the inset of Fig. 3a, demonstrating a good reproducibility.

## Discussion

Now we would like to discuss the origin of *M*_rob_. It is well known that, in antiferromagnets such as *α*-Fe_2_O_3_, spin canting due to the Dzyaloshinskii-Moriya (DM) interaction induces weak ferromagnetism^17^,^18^,^19^. However, such spin canting should not occur in the AIAO order, because the DM interactions geometrically cancel out in every tetrahedron. On the other hand, it is suggested that the weak ferromagnetism of Cd_2_Os_2_O_7_ is not a bulk property because its magnitude shows a significant sample dependence^10^. In fact, our second crystal B has a larger *M*_rob_ than the first crystal A, as compared in Fig. 2d. Thus, we think that *M*_rob_ originates from MDWs; the sample dependence must be due to a difference in the density of crystalline defects that pin down MDWs. The magnitude of *M*_rob_ is reasonably explained by the observed density of MDWs^14^ (Supplementary Section 2).

Then the key question is whether the MDW magnetism can explain the observed feature of *M*_rob_. In general, the spin structure of MDWs in an antiferromagnet without magnetic anisotropy is similar to that of a ferromagnet, in which a gradual rotation of magnetic moments occurs. These MDWs can carry uncompensated moments but they must be easily flipped by external fields^5^,^20^. In contrast, an antiferromagnet with a large anisotropy can generate MDWs of monolayer thickness at the anti-phase boundary^21^. This is actually the case for Cd_2_Os_2_O_7_ where a large easy axis anisotropy of 6.8 meV along the local threefold rotation axis of the trigonally distorted OsO_6_ octahedron stabilizes the AIAO order^22^. Probably, monolayer MDWs carrying Ising-like, uncompensated moments are generated in Cd_2_Os_2_O_7_. However, it is not trivial whether these uncompensated moments behave cooperatively to show ferromagnetic behavior. For example, in Na_2_Ba_3_[Fe_3_(C_2_O_4_)_6_][*A*(C_2_O_4_)_3_] (*A* = Sn, Zr) which exhibits an anisotropic antiferromagnetic order analogous to the AIAO order on a distorted kagome lattice, uncompensated moments appear at the monolayer MDWs and behave as uncorrelated quasi-free moments^23^. A specific nature of the AIAO order must be important as mentioned in the next paragraph.

Since Cd_2_Os_2_O_7_ has a cubic symmetry, the {001}, {110} and {111} planes may give stable interfaces. First we focus on the {001} MDW. In terms of the classical spin model, when AIAO and AOAI domains meet at an (001) plane, an interface layer consisting of tetrahedra having the 2-in/2-out spin configuration is always formed, as illustrated in Fig. 3c (Supplementary Section 3 and Supplementary Fig. 1). Note that each 2-in/2-out tetrahedron possesses a net moment perpendicular to the plane, and all of them align in the same direction at one MDW. This “ferromagnetic” moment in total can hardly flip by external *H*, because it is forced to align by the adjacent antiferromagnetic AIAO/AOAI domains and also because the Zeeman energy is minimal due to the monolayer thickness. To flip this, the 2-in/2-out layer must be shifted up (Fig. 3d) or down by one layer. Such a shift of the MDW must be suppressed by the large uniaxial magnetic anisotropy at low temperatures, while becomes possible at high temperatures close to *T*_N_ where the magnetic order is “soft”. For this reason, the uncompensated magnetic moment of the {001} MDW should behave as such a robust ferromagnetic moment as *M*_rob_ when they are aligned by *H*_FC_.

Concerning the other types of MDWs, the interface layers of the {110} and {111} MDWs contain tetrahedra having the 3-in/1-out or 1-out/3-in spin configuration, which also possess uncompensated magnetic moments as in the 2-in/2-out tetrahedron (Supplementary Fig. 1). However, these moments in the {110} and {111} MDWs are expected to behave as uncorrelated quasi-free moments without a ferroic alignment. Additional magnetizations probably corresponding to these free moments are actually observed at low temperatures below 20 K (Supplementary Section 5 and Supplementary Fig. 3).

In an actual crystal, it is expected that MDWs of various types and orientations are randomly generated when it is cooled through *T*_N_ in the absence of external fields. However, most of them tend to annihilate each other at low temperatures because they are energetically unfavorable, while some of them may survive by being trapped at crystalline defects; the density of the MDWs depends on the density of the crystalline defects. Possibly, a pinning of MDWs occurs at point defects like vacancies or cracks which suppress spin flipping required for the propagation of MDWs. On the other hand, the application of magnetic field upon cooling may reduce the density of the MDWs: since the two kinds of domains, AIAO and AOAI, should have different magnetizations under the 〈111〉 magnetic field^13^, either kind of domains having larger *M* are stabilized and dominate, which is in fact observed experimentally^14^. An additional effect of FC is to polarize the uncompensated moments at the MDWs. In ZFC, the ferromagnetic moments of the {001} MDWs cancel each other out, while, in FC, the gain in Zeeman energy stabilizes MDWs that have ferromagnetic components along the applied field, resulting in a net ferromagnetic moment. Field effects on the other types of MDWs may be negligible as the uncompensated spins behave as nearly free moments. Thus, the observed enhancement in magnetizations in the FC curve in Fig. 1c is ascribed to the polarization of the ferromagnetic {001} MDWs.

Next, we show evidence of electron conductivity in the {001} MDWs. Figure 1d compares two *ρ* curves measured at FC and ZFC. Although the bulk becomes insulating below *T*_N_, as evidenced by the observation of large optical gaps^24^,^25^, there remains a finite conductivity at the lowest temperature, which has been ascribed to conducting MDWs^14^,^17^. Note that the *ρ* of FC is apparently larger than the *ρ* of ZFC below 100 K, which may be due to the decrease of the conducting MDW density in the FC process^14^; the contribution of sparse conducting MDWs is discernible only at low temperatures where the bulk *ρ* becomes large enough. A similar variance in *ρ* between FC and ZFC experiments is observed in Nd_2_Ir_2_O_7_^15^ in which the presence of conducting MDWs has been well established^16^.

**Figure 4:**
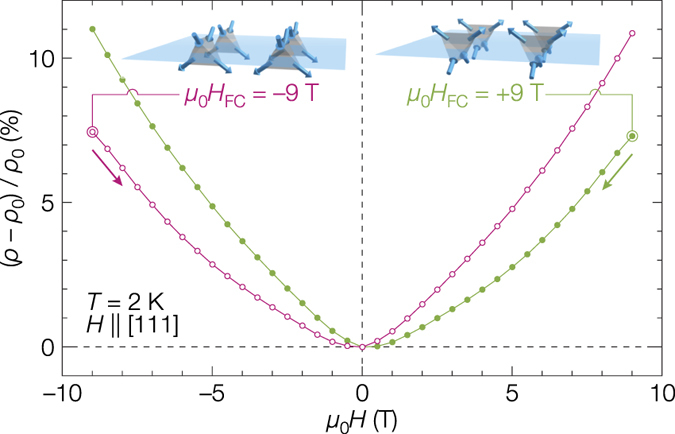
Magnetoresistance including an unusual odd-symmetric contribution. The *ρ*_0_ is the resistivity at zero magnetic field. The two curves were obtained at 2 K after cooling under the magnetic fields of *μ*_0_*H*_FC_ = +9 and −9 T along the [111] direction. Note that the sign of the odd-symmetric contribution is reversed depending on the sign of *H*_FC_. The insets illustrate the polarized spin structures of the (001) MDW expected for the positive and negative *H*_FC_.

An interplay between the ferromagnetism and electron conductivity at the MDWs is observed in the magnetoresistance shown in Fig. 4. There is an unusual odd-symmetric contribution superimposed on a conventional positive magnetoresistance in each curve. This odd-symmetric contribution is obviously different from the Hall resistivity because its sign is reversed with the sign of *H*_FC_. It is plausible that the robust ferromagnetism of the {001} MDWs affects conducting electrons in the vicinity of the MDWs. In such MDWs with both robust ferromagnetic moments and conducting electrons, one would expect an anomalous Hall effect or a spin-polarized electric current.

Finally, we would like to comment briefly on the relation between Cd_2_Os_2_O_7_ and *Ln*_2_Ir_2_O_7_, both of which exhibit similar MI transitions accompanied by the AIAO magnetic order and also possess similar MDWs. The basic electronic structures of the two compounds are quite different because of the different electron filling; 5*d*^3^ and 5*d*^5^ for Os^5+^ and Ir^4+^ ions, respectively^26^,^27^. The origin of the conducting MDWs in Nd_2_Ir_2_O_7_^15^,^16^ is suggested to be the topological interface state arising from the Weyl points of opposite chiralities in the Weyl semimetal^27^,^28^,^29^. Possibly, a similar Weyl semimetallic state exist in Cd_2_Os_2_O_7_^17^. Otherwise, there is an alternative reason that is common to the two compounds.

To summarize, we have discovered the unique ferromagnetism in the MDWs of the AIAO order in Cd_2_Os_2_O_7_. It is robust at low temperatures but can be easily flipped by a weak *H*_FC_ upon cooling across *T*_N_. The origin is ascribed to uncompensated magnetic moments in 2-in/2-out tetrahedron layers at the {001} MDW. Moreover, the electrical conductivity coexists in the MDWs, which is affected by the polarization of the robust ferromagnetism. These unique magnetic and electric properties of the MDWs in the AIAO order are not only interesting in materials science, but also important in the domain wall nanoelectronics. It would be possible to produce, for example, such a novel microscopic magnetic memory device as proposed in Supplementary Section 6 and Supplementary Fig. 4.

## Methods

### Sample preparation

Single crystals of Cd_2_Os_2_O_7_ were grown by the chemical transport technique described in ref. 10. First, a polycrystalline sample was prepared from CdO and elemental osmium in ratio of 1.05:1 and 1.1:1 for batches A and B, respectively; the excess CdO was required to compensate a loss of Cd during the reaction. A mixture was sealed in an evacuated quartz tube with oxygen supplier Ag_2_O_2_, heated up to 1073 K and kept for 2 days. The obtained polycrystalline pellet was put in an evacuated quartz tube and placed in a temperature gradient of 1040–1200 K for 10 days. Several single crystals of the octahedral shape up to 1 mm^3^ volume were obtained at the middle of the tube together with OsO_2_ crystals at the lower-temperature side. One of key tricks for better crystal growth is to clean the tube carefully before the reaction. Two crystals were selected from batches A and B and used in the measurements: crystals A and B from batches A and B weigh 1.970 and 0.994 mg, respectively.

### Magnetization measurements

The magnetizations of crystals A and B were measured in the temperature range of 2–350 K and in the field range between ±7 T in a SQUID magnetometer (MPMS3, Quantum Design). The vibrating sample magnetometer (VSM) mode allowed rapid measurements with a high accuracy of 10^−9^ emu and made it possible to obtain reliable data on one tiny single crystal.

In order to detect small ferromagnetic magnetizations from MDWs, which are superimposed on a much larger linear response from the bulk, it is crucial to know the exact magnitude of magnetic field near zero field at the sample position in the superconducting magnet of MPMS3. In general, there is always an inevitable remanent field of ~20 Oe left inside a superconducting magnet even if one sets “apparent” field *H*′ zero because of a trapped magnetic flux. This remanent field makes it difficult to obtain a true *M*-*H* curve, as it changes the direction upon changing *H*′ across zero; a minus (plus) remanent field is left when *H*′ is reduced to zero from plus (minus) fields. We have carefully estimated the remanent field as a function of *H*′ by measuring a reference paramagnetic sample of elemental palladium and corrected the actual applied magnetic field on a sample.

Most magnetization measurements were carried out on crystal A after cooling from above *T*_N_ to the measurement temperatures under a magnetic field of +7 T along the [111] direction, if not particularly mentioned. We have also examined several crystals and obtained essentially the same results except for the magnitude of the weak ferromagnetic moments; the variation is probably relevant to the different density or distribution of MDWs pinned by crystalline defects.

### Transport measurements

Resistivity was measured on crystal B at temperatures between 2 and 300 K in magnetic fields up to ±9 T in a Physical Property Measurement System (Quantum Design). A standard four-probe method was employed with electrical current running along the [1
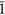
0] direction perpendicular to a magnetic field applied along the [111] direction.

### Illustration

Figures for crystal structure and spin arrangements were rendered using VESTA^30^.

## Additional Information

**How to cite this article:** Hirose, H. T. *et al*. Robust ferromagnetism carried by antiferromagnetic domain walls. *Sci. Rep.* **7**, 42440; doi: 10.1038/srep42440 (2017).

**Publisher's note:** Springer Nature remains neutral with regard to jurisdictional claims in published maps and institutional affiliations.

## Supplementary Material

Supplementary Information
