# Supplementary material for: Robust ferromagnetism carried by antiferromagnetic domain walls
Source: Sci Rep. 2017 Feb 14;7:42440. doi: 10.1038/srep42440 (PMC5308413; doi:10.1038/srep42440)
Supplement: Supplementary Information [file srep42440-s1.pdf]

Supplementary Information
Robust ferromagnetism carried by antiferromagnetic domain walls

Hishiro T. Hirose[1*], Jun-ichi Yamaura[2], & Zenji Hiroi[1*]

[1]Institute for Solid State Physics, University of Tokyo, Kashiwa, Chiba 277-8581, Japan
[2]Material Research Center for Element Strategy, Tokyo Institute of Technology, Yokohama, Kanagawa 226-8503, Japan
*e-mail: hishirose@gmail.com, hiroi@issp.u-tokyo.ac.jp

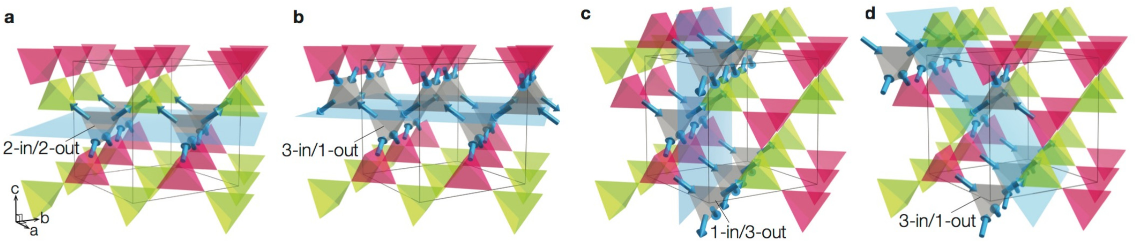

Figure S1. Various local structures of MDWs. Spin structures of (001) MDWs consisting of only 2-in/2-out tetrahedra (**a**) and only 3-in/1-out tetrahedra (**b**). Spin structures of (110) (**c**) and (111) MDWs (**d**) consisting of 3-in/1-out tetrahedra.

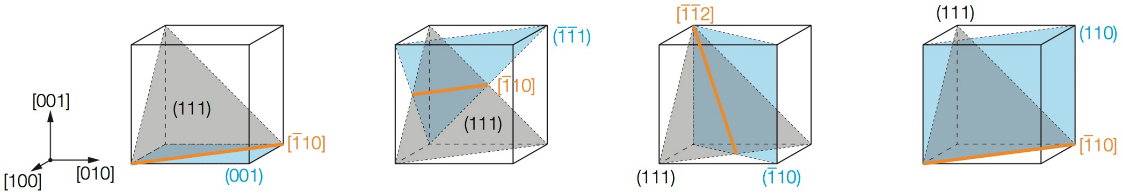

Figure S2. Schematic representations of MDWs (cyan sections) observed on the (111) facet of a crystal (gray section). The edges of the (001), (111), (−110), and (110) planes are [−110], [−110], [−1−12], [−110], respectively, as shown by orange lines.

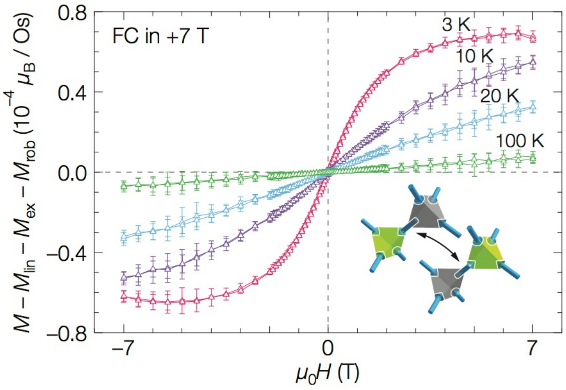

Figure S3. Magnetization curves at low temperatures with the magnetic field applied along the [111] direction after subtracting M_{lin}, M_{ex}, and M_{rob}. They were measured after cooling from a high temperature above T_N down to the temperatures under +7 T. $M - M_{ex} - M_{rob}$ can be well fitted by the sum of a linear term M_{lin} and a Brillouin function that is denoted as M_{free}; in order to estimate M_{free} correctly, all the M-H curves are fitted simultaneously using a Brillouin function of the identical weight. The inset illustrates two degenerate states for a pair of connected tetrahedra with 4–0/3–1 and 3–1/4–0 spin configurations which are realized at the {110} and {111} MDWs. The spins at the bridging site which can flip may be the origin of M_{free}.

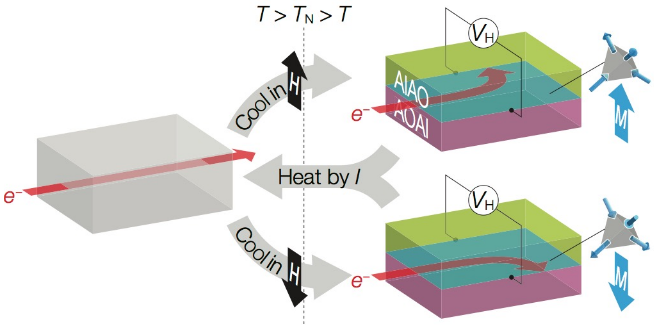

Figure S4. Conceptual representation of a microscopic magnetic memory device utilizing the robust ferromagnetic moment of the (001) MDW of the AIAO order for information storage. In the writing action, a crystal containing a (001) MDW at a low temperature below T_N is instantaneously heated above T_N by a pulse current and immediately cooled in a small magnetic field so that the ferromagnetic moment of the MDW align up or down along the field. In the reading action, a Hall voltage, that is induced by conduction electrons (e^-) confined around the MDW turning right or left depending on the directions of the ferromagnetic moments due to the anomalous Hall effect, is measured to know the memory.

S1. Ferromagnetic component above T_N: M_{ex}

$Cd_2Os_2O_7$ shows the ferromagnetic component M_{ex} which is present above T_N (Fig. 2b) and is almost temperature-independent upon cooling across T_N, in addition to the linear component M_{lin} from a paramagnetic or an antiferromagnetic state of bulk and the robust ferromagnetic component M_{rob} originated in the {001} MDWs. The M_{ex} of crystal A saturates already at 1 T to a small value of 6.7×10^{-5} μ_B/Os, which corresponds to ~0.05% of the magnetic moment of Os (μ_{Os} = 1–1.5 μ_B; Yamauchi, I. & Takigawa, M. in preparation), even above room temperature. Hence it must come from a ferromagnetic moment, though the magnetic hysteresis is almost absent. We have examined several crystals and always observed similar M_{ex}. The origin of M_{ex} is still unclear, but must be irrelevant to the AIAO order. We speculate that the M_{ex} is due to a tiny amount of ferromagnetic inclusion or a ferromagnetic layer formed on the crystal surface[1].

S2. Estimation of the MDW density from M_{rob}

An average separation d of MDWs on the (111) surface is estimated from the observed value of $M_{rob}(0) \sim 10^{-4}$ μ_B / Os. Provided that the entire $M_{rob}(0)$ along the [111] direction in crystal A comes from only (001) MDWs having the 2-in/2-out structure, the d is calculated by

$$d = a\mu_{Os} / \left[2\sqrt{6}M_{rob}(0)\right],$$

where a is the lattice constant [10.1618(8) Å]. Then, d is calculated as 2–3 μm. This value is considerably smaller than the actually observed value of ~20 μm by the circular polarized resonant X-ray diffraction imaging technique[2]. However, the surface density of MDWs can be smaller than the bulk density as a result of annihilation of small domains at the surface. Moreover, there is a significant sample dependence in the density. Thus, we think that our MDW model reasonably account for the magnitude of the observed M_{rob}.

S3. Stability of MDWs

The local structures of MDWs are considered in terms of the classical spin model. In the extreme limit of the strong Ising anisotropy[3], four magnetic moments on the vertices of tetrahedron can point only in or out to the center of the tetrahedron. As a result, three types of spin configurations are possible: 4-in or 4-out (denoted as 4–0), 3-in/1-out or 1-in/3-out (3–1), and 2-in/2-out (2–2). The energies of these configurations are given as $E_{40} = -6J_{eff}$, $E_{31} = 0$, and $E_{22} = 2J_{eff}$, where J_{eff} is the nearest-neighbor effective antiferromagnetic interaction ($J_{eff} > 0$). The stability between these MDWs can be compared in terms of these energies.

The energy of a MDW E_{MDW} defined as the energy cost per area is calculated as $2(E_{22} - E_{40})/a^2 = 16J_{eff}/a^2$ for a (001) MDW consisting of only 2–2 tetrahedra (Fig. S1a) On the other hand, the E_{MDW} of a (001) MDW consisting of only 3–1 tetrahedra (Fig. S1b) is $24J_{eff}/a^2$. More complex (001) MDWs containing both 2–2 and 3–1 tetrahedra apparently take larger energies than $16J_{eff}/a^2$. Therefore, the 2–2 structure of Fig. S1a is the most stable for the {001} MDWs. Similarly, for {110} and {111} MDWs, domain walls containing only 3–1 tetrahedra as depicted in Fig. S1c and d are most stable with E_{MDW} ~$17.0J_{eff}/a^2$ and ~$13.9J_{eff}/a^2$, respectively. Therefore, the three kinds of MDWs can coexist in a crystal, because their E_{MDW} values are relatively close to each other. In addition, the potential barriers between them and also a pinning by defects may help the coexistence.

S4. MDWs observed on the (111) crystal surface

The experimentally visualized domain pattern on a (111) facet of a single crystal provides important information about the coexistence of MDWs: two kinds of rectilinear MDWs running along the <110> and <112> direction are observed[2]. Figure S2 shows how the three kinds of MDWs appear on the (111) facet. Both the {001} and {111} MDWs should appear as <110> edges, while the {110} MDWs as either <110> or <112> edges. Therefore, it is clear that the{110} MDWs are generated and that at least either the {001} or {111} MDWs exist. Taking into account the fact that only the {001} MDWs can explain the M_{rob}, the {001} and {110} MDWs must coexist and the {111} MDWs are not excluded.

S5. Magnetic properties of the {110} and {111} MDWs

The {110} and {111} MDWs also carry uncompensated magnetic moments as they contain tetrahedra with the 3–1 spin configuration. Note that the interface layers of these MDWs consist of pairs of 4–0 and 3–1 tetrahedra as shown in the inset of Fig. S3. Two states, 4–0/3–1 and 3–1/4–0, in a pair have the same energy and are interchanged by flipping the bridging magnetic moment. Consequently, the uncompensated magnetic moments in the {110} and {111} MDWs are expected to behave as uncorrelated quasi-free moments, which is in sharp contrast to the cooperative ferromagnetic behavior of those in the {001} MDWs.

Probably corresponding to these contributions from the {110} and {111} MDWs, an additional magnetic component was observed at low temperatures. One evidence is the Curie-like upturn appearing in the T dependence of M/H towards $T = 0$ in Fig. 1c. Moreover, as shown in Fig. S3, the magnetization M_{free} left after the subtraction of M_{lin}, M_{ex}, and M_{rob} gradually grows upon cooling below 100 K and almost saturates at 3 K in 7 T. The lowest-temperature M–H curve can be fitted well by the Brillouin function, indicating the presence of non-interacting free magnetic moments; the Curie-like upturn must also come from this M_{free}. The magnitude of M_{free} is as small as 7×10^{-5} μ_B/Os and happens to be nearly equal to that of M_{rob}. Thus, it is likely that the M_{free} also comes from uncompensated moments at the {110} and {111} MDWs. However, an interesting question is why they do not freeze at such low temperatures. There is an energy barrier as large as 80 K due to the strong magnetic anisotropy[3] for a magnetic moment at the bridging site in the 3–1/4–0 pair to flip. Thus, the spin should freeze in the classical picture. Possibly, a certain quantum effect play a role in the flipping process. In fact, a quantum tunneling effect is pointed out to understand the quasi-free spins of MDWs in the distorted kagome antiferromagnets $Na_2Ba_3[Fe_3(C_2O_4)_6][A(C_2O_4)_3]$ (A = Sn, Zr)[4]. This may be also the case for Ising spins in the spin-ice system on the pyrochlore lattice, where spin flipping occurs at much lower temperatures compared to the magnetic interaction energy[5].

S6. Possible application in the domain wall nanoelectronics

The {001} MDW found in this study is atomically thin and possesses a robust ferromagnetic moment together with electrical conductivity. The direction of the ferromagnetic moment can be controlled by a small field upon cooling, and the magnitude is reproducible in repetition, as demonstrated in Fig. 3a. Taking all these unique features of the MDW together, it would be possible to design a novel electronic device used in the domain wall nanoelectronics. Here we propose an example of such a novel magnetic memory device as illustrated in Fig. S4.

Let us assume a microscopic crystal containing a single or a set of parallelly aligned (001) MDWs. The direction of the robust ferromagnetic moments is used to store information; up ('0') or down ('1'). In the writing process, the crystal is heated instantaneously up to above T_N by Joule heating using a pulsed electrical current and is immediately cooled in a small magnetic field generated by a mini coil, so that the robust ferromagnetic moment polarizes along the field. Once cooled down to a low enough temperature, the memory is kept robust against external fields. In the reading process, the anomalous Hall effect is used to detect the up or down polarization of the MDW magnetization. Since the MDW is atomically thin, a large response in the Hall voltage is expected even for a small polarization. In addition, a higher-density integration could be achieved because domain walls in antiferroic orders are more stable for miniaturization than those in ferroic orders. Therefore, employing the MDWs of the AIAO order would provide us with a novel route to a new type of magnetic memory to be used in the domain wall nano electronics in the future. One practical problem to get over for this is to find a more appropriate material that has a higher T_N above room temperature to the AIAO order and is easy to handle in the manufacturing process than $Cd_2Os_2O_7$. We believe that there is a chance to discover such a compound or other type of Ising antiferromagnets that carry useful MDWs in nature.

References of Supplementary Information

1. Taniuchi, T. *et al.* Imaging of room-temperature ferromagnetic nano-domains at the surface of a non-magnetic oxide. *Nat. Commun.* **10,** 1038 (2016).
2. Tardif, S. *et al.* All-In-All-Out Magnetic Domains: X-Ray Diffraction Imaging and Magnetic Field Control. *Phys. Rev. Lett.* **114,** 147205 (2015).
3. Bogdanov, N. A., Maurice, R., Rousochatzakis, I., Brink, J. van den & Hozoi, L. Magnetic State of Pyrochlore $Cd_2Os_2O_7$

Emerging from Strong Competition of Ligand Distortions and Longer-Range Crystalline Anisotropy. *Phys. Rev. Lett.* **110,** 127206 (2013).

4. Lhotel, E. *et al.* Domain-Wall Spin Dynamics in Kagome Antiferromagnets. *Phys. Rev. Lett.* **107,** 257205 (2011).
5. Matsuhira, K., Hinatsu, Y., Tenya, K. & Sakakibara, T. Low temperature magnetic properties of frustrated pyrochlore ferromagnets $Ho_2Sn_2O_7$ and $Ho_2Ti_2O_7$. *J. Phys. Condens. Matter* **12,** L649–L656 (2000).
